# A review on fish‐borne zoonotic parasites in Iran

**DOI:** 10.1002/vms3.981

**Published:** 2022-10-21

**Authors:** Nasser Hajipour, Hadi Valizadeh, Jennifer Ketzis

**Affiliations:** ^1^ Faculty of Veterinary Medicine Department of Pathobiology, University of Tabriz Tabriz Iran; ^2^ Faculty of Veterinary Medicine Department of Food Hygiene and Aquatic, University of Tabriz Tabriz Iran; ^3^ Biomedical Sciences Ross University School of Veterinary Medicine, Basseterre, St Kitts, West Indies

**Keywords:** fish‐borne, food safety, Iran, parasite, zoonotic

## Abstract

**Background:**

Fish is a great nutritious food and provides quality protein and a variety of vitamins and minerals. This contributes significantly to the economy and food security in Iran. However, there are safety concerns related to the presence of zoonotic parasites.

**Objectives:**

The objective of this study is, therefore, to review fish‐borne zoonotic parasites in Iran.

**Methods:**

Keywords such as fish‐borne, parasites, zoonotic, Iran, and some names of fish‐borne zoonotic parasites were searched in databases including PubMed, Science Direct, Elsevier, SID, Magiran, Irandoc, Google Scholar and the World Health Organization.

**Results:**

The most common fish‐borne parasites with zoonotic potential identified in reports in the literature were the protozoa *Balantidium* spp., *Myxobolus* spp. and *Sarcosystis* sp.; the trematodes *Heterophyes heterophyes* and *Clinostomum complanatum*; the cestodes *Ligula intestinalis* and *Diphyllobothrium latum*; the nematodes *Pseudoterranova* sp., *Anisakis* spp., *Contracaecum* spp., *Raphidascar*is spp., *Eustrongylides* spp. and *Capillaria* sp.; and the acanthocephal *Corynosoma* spp.

**Conclusions:**

The potential risk factors for the transmission of fish‐borne zoonotic parasites to humans are consumption of raw or undercooked infected fish, contact with contaminated water and contact with infected fish. There is a need for epidemiological surveillance of fish for parasites with zoonotic potential and of occurrence of infections in humans to better understand the public health significance and design prevention programs.

## Graphical abstract

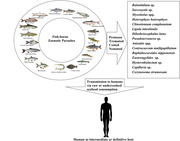

## 1 INTRODUCTION

The aquaculture industry including fish farming is one of the developing industries in the area of food production. Because fish meat is an important source of nutrients, especially essential fatty acids that are in optimal quantities for human needs, demand for fish meat is continuously increasing (Ljubojevic et al., 2015a, 2015b). Seafood consumption per capita in Iran reached 13.3 kg in the year ending March 2021, indicating a 56% increase compared with 8.5 kg in 2013 (Adeli, 2013). While fish can be an important source of nutrients, consumption of raw or inadequately cooked fish can be a major public health concern due to is fish‐borne zoonotic parasites (FBZPs) (Chai et al., 2005). Consumption of raw fish such as sushi and sashimi, commonly found in Japan's national dishes, as well as the culinary tradition of consumption of marinated or raw fish in European countries such as Italy are a significant source of human infections with FBZP (Yorimitsu et al., 2013). The World Health Organization (WHO) estimates approximately 56 million cases of parasite infections associated with the consumption of raw or undercooked fish or fish products but worldwide the number of people at risk, including those in developed countries, is more than half a billion (Santos & Howgate, 2011; WHO, 1995, 2004, 2012). Recent official statistics suggest that approximately 1.5 million people in Korea, 6 million people in China and over 5 million in Thailand are infected with FBZPs including liver flukes such as *Clonorchis sinensis* and *Opisthorchis viverrini* (Chai et al., 2005; Chai, 2005).

In developed countries, people usually have information about meat‐borne zoonosis such as trichinellosis and cysticercosis, while far fewer are acquainted with fish‐borne parasitic zoonoses like opisthorchiasis, intestinal trematodiasis, anisakiasis or diphyllobothriasis. Yet these zoonotic parasites from fish are responsible for large numbers of human infections (Chai et al., 2005). Although epidemiological data are scarce, the prevalence and species diversity of FBZPs in Iran suggest that these parasites are also an important national public health problem (Pazooki & Masoumian, 2012; Rahmati et al., 2020). The main goals of this paper are to describe the most important parasitic hazards present in fish meat in Iran, to bring attention to the need for epidemiological studies on fish infections and human infections with zoonotic parasites and to highlight the need for increased public awareness concerning the risks of consuming incorrectly prepared fish meat.

## 2 METHODS

A systematic review of the literature was performed using online databases from various fields and in Iran from 1981 to 2020. Databases included PubMed, Science Direct, Elsevier, SID, Magiran, Irandoc, Google Scholar and the World Health Organization. Key words used included: fish‐borne zoonotic parasites, such as: ‘*Balantidium*’, ‘*Sarcocystis*’, ‘*Myxobolus*’, ‘*Heterophyes*’, ‘*Clonorchis*’, ‘*Opisthorchis*’, ‘*Diphyllobothrium*’, ‘*Ligula*’, ‘*Anisakis*’, ‘*Contracaecum*’, ‘*Raphidascaris*’, ‘*Eustrongylides*’, ‘*Hysterothylacium*’, ‘*Capillaria*’ and ‘*Corynosoma*’. As well as, to access the names of fish‐borne zoonotic parasites, was used an article published by Shamsi (2019). Grey literature such as theses, conference presentations and abstracts and articles published in national and international peer reviewed journals were included. Based on the principles of systematic review studies, non‐related documents were excluded and, after screening the publications, all documents whose contexts were in concordance with the aim of the present study were selected for final review. Primary reasons for exclusion included not about fish in Iran and no information on FBZPs. For the documents reviewed, data extracted were name of parasite(s), fish species, number of examined, percentage of infection, infected organ(s), methods performed and study area(s). All data were summarised in tabular form (Tables 1, 2, 3, 4, 5).

**TABLE 1:** Fish‐borne zoonotic protozoa in Iran

**Row**	**Parasites**	**Fish species**	**No. exam**.	**Prevalence (%)**	**Infected organs**	**Method**	**Lakes, river/area study**	**References**
1	*Balantidium* sp.	*Barbus sharpeyi*	20	25	Intestine	N	Shadegan and Sosangerd city	Rahdar et al. (2012)
2	*Balantidium* sp.	*B. grypus*, *B. sharpeyi*,	189, 67	ND	Intestine	N	Karun, Karkheh, Shadegan/Khuzestan Province	Seyed Mortezaei et al. (2008a)
3	*Balantidium* sp.	*B. grypus*, *B. sharpeyi*	189, 67	ND	Intestine	N	Karun, Karkheh, Shadegan/Khuzestan Province	Masoumain et al. (2007a)
4	*Balantidium* sp.	*B. sharpeyi*	N	36.5	Intestine	N	Shadegan/Khuzestan Province	Mesbah (2006)
5	*Balantidium* sp.	*B. sharpeyi*, *B. luteus*	146, 198	ND	Intestine	N	Wetlands of Khuzestan Province	(Seyed Mortezaei and Abbasi (2001)
6	*Balantidium* sp.	*B. sharpeyi*, *B. grypus*, *Planiliza abu*	ND	ND	Intestine	N	Hoor‐Alazim Lagoon/Khuzestan Province	Moghainemi (1996)
7	*B. ctenopharyngodoni*	*Ctenopharyngodon idella*	ND	ND	Intestine	N	Caspian Sea	Saeedi (1994)
8	*B. ctenopharyngodoni*	*Ctenopharyngodon idella*	ND	ND	Intestine	N	Hamoun Lake/Sistan and Baluchestan Province	Molnar and Baska (1993)
9	*Sarcosystis* sp.	*Barbus sharpeyi*	20	15	Muscle	D	Shadegan and sosangerd/Khuzestan Province	Rahdar et al. (2012)
10	*Myxobolus saidovi*	*C. trutta*	ND	ND	Gills	N	Gheshlagh (Vahdat) Reservoir, Kurdistan Province	Bozorgnia et al. (2012)
11	*M. buckei*	*Capoeta damascina*	109	9	Spinal cord	N	Halil‐Rud River, Kerman Province	Nazari Chamak et al. (2009)
12	*M. karelicus*	*C. damascina*	109	39.5	Intestine	N		
13	*M. cristatus*	*C. damascina*	109	59.5	Gills	N		
14	*M. musayevi*	*C. damascina*	109	60	Gills	N		
15	*M. samgoricus*	*C. damascina*	109	58	Fins	N		
16	*M. suturalis*	*C. damascina*	109	11	Heart, Muscle	N		
17	*M. varicorhini*	*C. damascina*	109	41	Liver, kidney, skin	N		
18	*M. musayevi*	*C. damascina*, C. *aculeata*	90	ND	Gills	N	Beheshtabad River, Chaharmahal and Bakhtiari Province	Raissy et al. (2009)
19	*M. karuni*, *M. presicus*	*B. grypus*, *B. sharpeyi*, *B. esocinus. B. barbulus*, *B. pectoralis*	296	33	Gills	N, H	Karoun, Karkheh Rivers and Shadgan Lagoon, Khuzestan Province	Masoumian et al. (2008)
20	*M. karuni*, *M. presicus*	*B. grypus*, *B. sharpeyi*, *B. esocinus*, *B. pectoralis*	189, 67, 8, 79	ND	Gills	N	Karoun, Karkheh Rivers and Shadgan Lagoon, Khuzestan Province	Seyed Mortezaei et al. (2008)
21	*M. nodulointestinalis*	*B. grypus*, *B. sharpeyi*, *B. pectoralis*	189, 67, 79	ND	Intestine	N		
22	*M. mesopotamiae*	*B. grypus*, *B. sharpeyi*	189, 67	ND	Fins	N		
23	*Myxobolus* sp.	*B. grypus*, *B. sharpeyi*,	189, 67	ND	Kidney	H		
24	*M. saidovi*	*C. capoeta grasilis*	ND	ND	Gills	N	Chalus River, Mazandaran Province	Miar et al. (2008)
25	*Myxobolus* sp.	*Carassius auratus gibelio*	23	17.3	Gills	N	Gandoman Lagoon, Chaharmahal and Bakhtiari Province	Raissy et al. (2007)
26	*M. cristatus*, *M. musayevi*	*C. capoeta*	68	20.5, 14.7	Gills	N	Zangbar Rud, Western Azerbaijan	Pazooki et al. (2007)
27	*Myxobolus persicus*, *M. karuni*	*B. grypus*, *B. sharpeyi*, *B. esocinus*, *B. pecturalis*	189, 67, 8, 79	ND	Gill	N	Karun, Karkheh, Shadegan/Khuzestan Province	Masoumain et al. (2007b)
28	*M. nodulointestinalis*	*B. grypus*, *B. sharpeyi*, *B. pecturalis*	189, 67, 79	ND	Intestine	H		
29	*M. mesopotamia*	*B. grypus*, *B. pecturalis*	189, 79	ND	Fins	H		
30	*M. pfiefferi*	*B. grypus*, *B. sharpeyi*, *B. esocinus*, *B. pecturalis*, *B. barbulus*	189, 67, 8, 79, 10	ND	Muscle	H		
31	*Myxobolus* sp.	*B. grypus*, *B. sharpeyi*,	189, 67	ND	Kidney	H		
32	*M. cristatus*	*Capoeta capoeta*, *C. aculeata*	50	ND	Gills	N	Zayandeh‐Rud River, Isfahan Province	Masoumain et al. (2007a,b)
33	*M. musayevi*	*Capoeta capoeta*	50	ND	Gills	N		
34	*M. saidovi*	*Alburnus maculatus*	50	ND	Gills	N		
35	*M. varicorhini*	*C. damascina*	50	ND	Fins	N		
36	*M. cristatus*	*C. capoeta grasilis*	30	Spring: 25 Summer: 57 Autumn: 58 Winter: 42	Gills	N	Sohrein Dam, Zanjan Province	Pazooki et al. (2005)
37	*M. musayevi*	*C. capoeta grasilis*	25	Spring: 37 Summer: 66 Autumn: 42 Winter: 25	Gills	N	Ghezel Uzoon, Zanjan Province	
38	*M. musayevi*	*C. capoeta grasilis*	22	Spring: 25 Summer: 57 Autumn: 50 Winter: 38	Gills	N	Sojasrood, Zanjan Province	
39	*M. musayevi*	*C. capoeta*	2	50	Gills	N	Aras and Mahabad Dams, Western Azerbaijan Province	Masoumian et al. (2003a)
40	*M. dispar*	*Aspius aspius taeniatus*	2	100	Gills	N		
41	*M. azerbaidjanicus*	*B. mursa*	125	2.4	Fins	N	Tajan and Zarem‐roud Rivers, Mazandaran Province	Masoumian et al. (2003b)
42	*M. kovali*	*B. mursa*	125	12	Fins	N		
43	*M. squamae*	*B. mursa*	125	5.6	Skin	N		
44	*M. tauricus*	*B. mursa*	125	12.8	Fins	N		
45	*M. rutili*	*B. mursa*	125	1.6	Fins	N		
46	*M. osmaniae*	*B. mursa*	125	1.6	Intestine	N		
47	*M. valdogeni*	*B.laceria*	21	9.5	Fins	N		
48	*M. musculi*	*B. capito*	16	6.2	Muscle	H		
49	*M. bramae*	*Rutilus frisii kutum*	10	ND	Gills	N	Tajan and Anzali Wetland, Gilan and Mazandaran Province	Masoumian and Pazoki (1998)
50	*M. ellipsoides*	*Alburnoides bipunctatus*	5	ND	Gills	N		
51	*M. minutus*	*Leuciscus cephalus*	11	ND	Gills	N		
52	*M. muelleri*	*Leuciscus cephalus*	11	ND	Gills	N		
53	*M. pavlovskyi*	*Hypophthalmichthys molitrix*	6	ND	Gills	N		
54	*M. pseudodispar*	*Chalcalburnus chalcoides*	5	ND	Gills	N		
55	*M. presicus*, *M. karuni*, *M. sharpeyi*, *M. nodulointestinalis*, *M. bulbocordis*, *M. iraanicus*	*B. sharpeyi*	50	22, 17, 22, 10, 10, 6	Gills, gills, intestine, heart, spleen	N, H	Hoor‐Elazim and Shadgan Marsh, River, Karon, Khuzestan Province	Masoumian and Pazooki (1999)
56	*M. presicus*, *M. karuni*, *M. nodulointestinalis*, *M. mesopotamiae*, *M. iraanicus*	*B. luteus*	59	20, 17, 15, 14, 16	Gills, gills, intestine, fins, spleen	N, H		
57	*M. presicus*, *M. karuni*, *M. mesopotamiae*, *M. iraanicus*	*B. grypus*	50	52, 52, 20, 6	Gills, fins	N, H	Hoor‐Elazim and Shadgan Marsh, River, Karon, Khuzestan Province	Masoumian and Pazooki (1999)
58	*M. shadgani*, *M. mesopotamiae*	*B. rajanorum*	18	22, 10	Gills, fins	N, H		
59	*M. molanari*, *M. mokhayeri*	*C. trutta*	8	25, 13	Gills, fins	N, H		
60	*M. nodulointestinalis*	*B. sharpeyi*, *B. luteus*	8, 9	9.6, 15.3	Intestine	N, H	Karun River, Khuzestan Province	Masoumian et al. (1996)
61	*M. iranicus*	*Barbus sharpeyi*, *B. grypus*	83, 50	ND	Spleen	H	Hoor‐Elazim and Shadgan Marsh, River, Karon, Khuzestan Province	Molnar and Pazoki (1996)
62	*M. mesopotamiae*	*B. luteus*, *B. rajanorum*	59, 18	ND	Spleen, fins	H		
63	*M. shadgani*	*B. rajanorum*	18	ND	Gills	N		
64	*M. sharpeyi*	*B. sharpeyi*	83	ND	Gills	N		
65	*M. mokhayeri*, *M. molnari*	*C. trutta*	8	12.5, 25	Fins, gills	N, H	Karoun River, Khuzestan Province	Baska and Masoumian (1996)

ND: not determined, N: necroscopy and microscopy, D: digestion, H: histological examinations.

**TABLE 2:** Fish‐borne zoonotic trematodes in Iran

**Row**	**Parasites**	**Fish species**	**No. exam**.	**Prevalence (%)**	**Infected organs**	**Method**	**Lakes/river/area study**	**References**
1	*Heterophyes heterophyes*	Fresh water fish	N	ND	Intestine	N	Iran	Long et al. (2017)
2	*Clinostomum complanatum*	*Alburnoides bipunctatus*, *Cobitis taenia*, *Squalius cephalus*, *Capoeta gracilis*	101, 101, 6, 103	7.9, 4.9, 16.6, 24.3	Abdominal cavity	N	Shiroud, Tajan and Gorganroud River/Southern Caspian Sea Basin	Aghlmandi et al. (2018)
3	*C. complanatum*	*Alburnus mossulensis*, *Capoeta damascina*, *Garra rufa*,*Squalius cephalus*	61, 98, 13, 66	5, 4.1, 15, 3	Muscles, operculum, fins	PCR	Gheshlagh River /Kurdistan Province	Maleki et al. (2018)
4	*C. complanatum*	*Cyprinus carpio*	480	14.79	Skin, fins, gills	N	Zarinerud River/West Azerbaijan Province	Rasouli and Purghasem (2016)
5	*C. complanatum*	*Aphanius dispar*	97	4.12	Fins, skin	N	Mehran River/Hormozgan Province	Gholami et al. (2011b)
6	*C. complanatum*	*C. capoeta gracilis*	230	A (28), W (36.7), S (23.3), Su (25)	Gills, operculum, muscles, fins	N	Sefid Roud River/Guilan Province	Ghazifard et al. (2011)
7	*C. complanatum*	*C. capoeta gracilis*	120	ND	Muscle, gills	N	Zarineh River/Miandoab	Azadikhah et al. (2010)
8	*C. complanatum*	*C. capoeta gracillis*	120	45.83	Gill, operculum, pharynx, muscle, skin	N	Shirood River/Caspian Sea	Sarang et al. (2007)
9	*C. complanatum*	*C. capoeta gracilis*	959	61.4	Gills, operculum, muscles, fins	N	Shirood RiverMazandaran	Roohi and Malek (2005)
10	*C. complanatum*	*C. capoeta gracilis*	112	47.3	Muscle	N	Shirood River/Caspian Sea	Malek and Moubedi (2001)
11	*C. complanatum*	*Alburnioides bipunctatus* *C. capoeta*, *Cobitis taenia*, *Leuciscus cephalus*	101, 103 101, 6	7.9, 24.3, 4.95, 16.6	Scales, gills	N	Shirood River/Caspian Sea	Shamsi et al. (1997)
12	*C. complanatum*	*C. capoeta*	112	Female (55.7, Male (34.1)	Muscle	N	Shirood River/Caspian Sea	Malek (1993)

ND: not determined, N: necroscopy and microscopy, PCR: polymerase chain reaction, A: autumn, W: winter, S: spring, Su: summer.

**TABLE 3:** Fish‐borne zoonotic cestodes in Iran

**Row**	**Parasites**	**Fish species**	**No. exam**.	**Prevalence (%)**	**Infected organs**	**Method**	**Lakes/river/area study**	**References**
1	*Ligula intestinalis*	*Macrostomum cyprinion*, *Capoeta damascina*	100	4, 11.11	Abdominal cavity	N	Seymareh River/Lorestan Province	Hoseinpour (2018)
2	*L. intestinalis*	*Abramis brama*	31	6.45	Abdominal cavity	N	Alagol lake/Golestan Province	Mazandarani et al. (2018)
3	*L. intestinalis*	*Alburnus filippi*	61	24.59	Abdominal cavity	N	Gorganroud/Golestan Province	
4	*L. intestinalis*	*Chalcalburnus* sp.	587	ND	Body cavity	N	Zarivar Lake/Kurdistan Province	Jalali and Barzegar (2006)
5	*L. intestinalis*	*Abramis brama orientalis*	60	26.66	Abdominal cavity	N	Caspian Sea/Babolsar, Mazandaran Province	Bozorgnia et al. (2016)
6	*L. intestinalis*	*Cyprinus carpio*	ND	ND	Intestine	N	Shah Cheraghi Dam/Semnan Province	Ebrahimian et al. (2014)
7	*L. intestinalis*	*Abramis brama*	120	67.5	Body cavity	N	Aras Reservoir/East Azerbaijan Province	Azadikhah et al. (2013)
8	*L. intestinalis*	*Chalcalburnus mossulensis*, *Pseudorasbora parva*, *Gambusia holbrooki*	ND	ND	Body cavity, intestine	N	Gheshlagh (Vahdat) Reservoir/Kurdistan Province	Bozorgnia et al. (2012)
9	*L. intestinalis*	*Chalcalburnus chalcoides*	65	83.08	Abdominal cavity	N	Saungar‐Dam/Giulan Province	Garedaghiand Mohammadi (2012)
10	*L. intestinalis*	*Abramis brama orientalis*	175	73	Abdominal cavity	N	Shores of Bandar Anzali/Giulan Province	Hayatbakhsh et al. (2012)
11	*L. intestinalis*	*Alburnus mossulensis*	1200	32	Abdominal cavity	N	Vahdat Dam/Kurdistan Province	Parsa et al. (2012)
12	*L. intestinalis*	*Aphanius dispar*	63	22.22	Abdominal cavity	N	Mehran River/Hormuzgan Province	Gholami et al. (2011a)
13	*L. intestinalis*	*Pseudorasbora parva*	108	13.60	Abdominal cavity	N	Chah‐nimeh reservoirs/Zabol, Sistan and baluchestan Province	Hosseini et al. (2011)
14	*L. intestinalis*	*Chalcalburnus mossulensis*	300	25	Abdominal cavity	N	Gheshlagh (Vahdat) Reservoir/Kurdistan Province	Parsa and Bahramian (2011)
15	*L. intestinalis*	*Alburnoides bipunctatus*	6	33.33	Intestine	N	Latian Reservoir Dam Lake/Tehran Province	Rahmati‐Holasoo et al. (2011)
16	*L. intestinalis*	*Pseudorasbora parva*	84	20.23	Abdominal cavity	ND	Chah‐nimeh reservoirs/Zabol, Sistan and baluchestan Province	Shahriari Moghadam and Ghanbari (2009)
17	*L. intestinalis*	Cyprinidae	310	ND	Abdominal cavity	N	Sattar Khan Dam/East Azerbaijan Province	Hajirostamloo (2009)
18	*L. intestinalis*	*Abramis brama orientalis*	256	45.7	Abdominal cavity	N	Aras Reservoir/East Azerbaijan Province	Nezafat Rahimabadi et al. (2008)
19	*L. intestinalis*	*Capoeta capoeta*, *Cyprinus carpio*, *Abramis brama*	166 7 23	21.8, 33.33, 38.46	Abdominal cavity	N	Ghalae Jough, Cheshme Souraya, Aras Dam, West Azerbaijan Province	Pazooki et al. (2007)
20	*L. intestinalis*	*Alburnus filippi*, *Alburnoides biponctatus*	1605	87.81, 80	Abdominal cavity	N	Sattar Khan Dam/East Azerbaijan Province	Mortazavi et al. (2005)
21	*L. intestinalis*	*Rutilus rutilus*	50	100	Abdominal cavity	N	Aras Reservoir/East Azerbaijan Province	Yousefi et al. (2005)
22	*L. intestinalis*	*Alburnus charousini*	106	35.8	Abdominal cavity	N	Shahid Modarres barrage/Kashmar, Razavi Khorasan Province	Pazooki and Aghlmandi (2001)
23	*L. intestinalis*	*Cyprinus carpio*	Case report	ND	Abdominal cavity	N	Ponds/West Azerbaijan Province	Abdi and Mobedi (2000)
24	*Dibothriocephalus latus* plerocercoid	*B. brachycephalus*	Case report	ND	Intestinal	N	Sefid‐Rood River/Guile	Mokhayer (1981)
25	*D. latus* plerocercoid	*B. sharpeyi*, *B. grypus*, *Planiliza abu*	516	2.32	Intestinal	N	Hoor‐Alazim Lagoon/Khuzestan Province	Moghainemi (1996)

ND: not determined, N: necroscopy and microscopy.

**TABLE 4:** Fish‐borne zoonotic nematodes in Iran

**Row**	**Parasites**	**Fish species**	**No. exam**	**Prevalence (%)**	**Infected organs**	**Method**	**Lakes/river/area study**	**References**
1	*Pseudoterranova* sp.	*Saurida tumbil*, *Tylosurus crocodilus crocodiles*	31, 34	ND	Intestine, Stomach, Muscle, Ovary Body cavity	N	Persian Gulf/Choebded, Boushehr and Dayyer ports	Dadar et al. (2016)
2	*Pseudoterranova* sp.	*Psettodes erumei*	97	4.1	Stomach	N	Persian Gulf/Market of Chabahar	Hosseini et al. (2013)
3	*Anisakis pegreffii*	*Mesopotamichthys sharpeyi*, *Barbus grypus*	200	6, 4	N	PCR	Shadegan Marsh of Khuzestan Province	Mohammadi et al. (2021)
4	*A. simplex*	*Cyprinus carpio*	36	2.78	Abdominal cavity	N	Caspian Sea, Bandar‐Torkaman	Taheri Mirghaed et al. (2019)
5	*A. pegreffii*	*Mesopotamichthys sharpeyi*, *Barbus grypus*	100, 100	6, 4	Intestine	N	Shadegan Wetland/Khuzestan Province	Mohammadi et al. (2018)
6	*A. simplex*	*Alosa caspia*	30	33.33	Intestine, Liver, Mesentery	N	Caspian Sea, Bandar‐Torkaman/Mazandaran Province	Mirghaed (2017)
7	*Anisakis* sp.	*Saurida tumbil*, *Nemipterus japonicas*, *Tylosurus crocodilus crocodiles*, *Carangoides armatus*	31, 37, 34, 37	ND	Abdominal cavity	N	Persian Gulf, Choebded, Boushehr and Dayyer ports	Dadar et al. (2016)
8	*Anisakis* sp.	*Acipenser persicus*	5	10	Internal organs	N	Shahid Rajaee Propagation and Rearing	Adel et al. (2016)
9	*A. simplex*	*Alosa saposchnikowii*	30	43.33	Intestine, Liver, Mesentery	N	Southeastern part of the Caspian Sea	Mazandarani et al. (2016)
10	*A. typica*	*Otolithes ruber*, *Psettodes erumei*, *Saurida tumbil*, *Scomberomorus commerson*	120, 120, 120, 120	0.84, 5, 4.17, 3.34	Abdominal cavity	N	Persian Gulf/Bandarabbas, Hormozgan	Shamsi et al. (2016)
11	*Anisakis* Larval type I	*Otolithes rubber*	212	ND	Abdominal cavity	N	Persian Gulf/Bandarabbas, Hormozgan Province	Shohreh and Ghadam (2016)
12	*Anisakis* sp.	*Scomberomorus commerson*, *Otolithes rubber*, *Acipenser persicus*	100, 60, 20	3, 10, 10	Abdominal cavity	N	Caspian Sea and Persian Gulf	Adel et al. (2015)
13	*Anisakis* sp.	*Otolithes rubber*	25	5	Abdominal cavity	N	Persian Gulf/Bandarabbas, Hormozgan Province	Ebrahim Zadeh Mosavi et al. (2015)
14	*Anisakis* sp.	*Acanthopagrus latus*	276	6.5	Abdominal cavity, Intestine, Liver, Mesentery	N	Bahmanshir, Arvand Rud, Hengam and Ahvaz Markets /Khuzestan Province	Rasouli (2015)
15	*Anisakis* sp.	*Scomberomorus commerson*, *Nemipterus japonicas*	25, 25	8, 8	Intestine, Stomach, Abdominal cavity	N	Persian Gulf/Bandarabbas, Hormozgan	Ebrahim Zadeh Mosavi et al. (2014)
16	*A. simplex*	*Sander lucioperca*	30	36.67	Abdominal cavity	N	Southeastern part of the Caspian Sea	Mazandarani et al. (2014)
17	*Anisakis* sp.	*Otolithes rubber*	60	10	Digestive system, Abdominal cavity	N	Persian Gulf/Ahvaz, Khuzestan Province	Adel et al. (2014)
18	*Anisakis* sp.	*Scomberomorus commerson*	100	13	Abdominal cavity, Muscle	N	Persian Gulf/Bandar Abbas	Adel et al. (2013b)
19	*Anisakis* sp.	*Cyprinus carpio*	50	4	Abdominal cavity	N	Aras Dam/West Azerbaijan Province	Khodadadi et al. (2013)
20	*Anisakis* sp.	*B. barbulus*	69	5.79	Digestive system	N	Karoon River/Ahvaz	Zahiri and Razijalali (2012)
21	*Anisakis* sp.	*Clupeonella grimmi*	252	0.4	Digestive system, Abdominal cavity and Gonads	N	Caspian Sea/Babolsar	Jeddy et al. (2012)
22	*Anisakis* sp.	*Cyprinio macrostomum*, *Mastacembelus mastacembellus*, *B. grypus*, *B. luteus*	11, 13, 53, 97	27.3, 15.39, 15.09, 6.18	Intestine	N	Zohreh/Khuzestan Province	Dadar et al. (2011)
23	*Anisakis* sp.	*Thunnus tonggol*	100	89	Stomach, Abdominal cavity, liver, testicle, spleen, intestinal contents	N	Persian Gulf/Bandarabbas, Hormozgan Province	Eslami et al. (2011)
24	*Anisakis* sp.	*B. grypus*	40	10	Digestive system, abdominal cavity	N	Karkheh River/Khuzestan Province	Mesbah et al. (2010)
25	*Anisakis* sp.	*Clupeonella grimmi*	136	0.7	Intestine	N	Caspian Sea/Babolsar	Ghayoumi et al. (2009)
26	*A. pegreffii*	*Saurida tumbil*	N	N	Abdominal cavity	PCR	Oman sea	Pahlavan et al. (2013)
27	*Anisakis* sp.	*Cyprinion carpio*, *B. grypus*, *B. sharpeyi*, *B. luteus*, *Silurus triostegus*, *B. pectoralis*, *Aspius vorax*	94, 75, 94, 175, 22, 8, 44	ND	Intestine	N	Hoor‐Shadegan/Khuzestan Province	Seyed Mortezei et al. (2008b)
28	*Anisakis* sp.	*B. brachycephalus*, *Neogobius bathybius*	10, 67	20, 25.4	Abdominal cavity, intestine	N	Southern shore of the Caspian Sea	Sattari et al. (2008)
29	*Anisakis* sp.	*Otolithes rubber*, *Parastromateus niger*, *Pomadasys kaakan*, *Lutjanus malabaricus*	80, 80, 80, 80	3.75, 7.5, 5, 43.6	Abdominal cavity	N	Persian Gulf/Ahvaz, Khuzestan Province	Peyghan et al. (2006)
30	*Anisakis* sp.	*Acipenser gueldenstaedti*, *Huso huso*	78, 6	5.56, 16.67	Abdominal cavity	N	Southwest of the Caspian Sea	Sattari and Mokhayer (2006)
31	*Anisakis* sp.	*Neogobius bathybius*, *Barbus capito*, *Chalcalburnus chalcoides*	24, 5, 51	8.6, 20, 7.8	Abdominal cavity	N	Anzali Wetland/Guiln Province	Sattari et al. (2005)
32	*Anisakis* sp.	*Epinephelus coioides*	80	1.25	Abdominal cavity	N	Persian Gulf/Ahvaz, Khuzestan Province	Peyghan et al. (2004)
33	*Anisakis* sp.	*Rutilus frissi kutum*, Neogobius melanostomus	14, 24	14, 16	Abdominal cavity, outer wall of the intestine	N	Caspian Sea	Saeedi and Fani saberi (2003)
34	*Anisakis* sp.	*Acipenser persicus*, *Acipenser gueldenstaedti*	261	8.19, 3.13	Abdominal cavity	N	Caspian Sea	Gorbani (2000)
35	*Anisakis* sp.	*Acipenser persicus*, *Acipenser gulden staedti*	126, 113	19.8, 13.3	Eye, liver, spleen, swim bladder	N	Caspian Sea/Golestan Province	Hajimoradloo and Ghorbani Nasrabadi (2003)
36	*Anisakis* sp.	*B. grypus*, *B. lateus*, *Cyprinus carpio*, *Liza abu*, *Aspius vorax*	701	6.84	Muscle, abdominal cavity	N	Atash, Sobhanieh, Al‐hai and Houfel/Khuzestan Province	Farahnak and Tabibi (2002)
37	*Anisakis* sp.	*Rutilus rutilus caspicus*	140	3.7	Abdominal cavity	N	Southeast of the Caspian Sea, Sari and Bandar‐Torkaman/Mazandaran Province	Masoumian et al. (2002)
38	*Anisakis* sp.	*Clupeonella grimmi*	490	N	Abdominal cavity	N	Caspian Sea	Shamsi et al. (1998a)
39	*Anisakis* sp.	*Clupeonella grimmi, C. cultriventris*	490, 605	1, 0.2	Gastrointestinal tract	N	Babolsar Mazandaran Province, Bandar Anzali, Gilan Province	Shamsi et al. (1998b)
40	*Anisakis* sp.	*Rutilus frisii katum*	140	1.4	Abdominal cavity	N	South Caspian Sea	Eslami and Kohneshahri (1978)
41	*Anisakis* sp.	*Euthynnus* spp., *Lucioperca lucioperca*	ND	75, 20	Abdominal cavity	N	Persian Gulf, Caspian Sea	Eslami and Mokhayer (1977)
42	*A. schapakovi*	*Huso huso*	ND	ND	Abdominal cavity	N	Caspian Sea	Ghargi and Pourgholam (1995)
43	*Contracaecum multipapillatum*	*Aphanius hormuzensis*, *Oreochromis niloticus*, *Tilapia galilaea*, *Lates niloticus*, *Hydrocynus forskalii*	119, 73, 712, 85, 35	17.22, 35.6, 0.14, 100, 82	Digestive system	N, PCR	Shur River/Hormuzgan basin in Southern Iran	Motamedi et al. (2019)
44	*Contracaecum* spp.	*Mesopotamichthys sharpeyi*, *Barbus grypus*	200	9	N	PCR	Shadegan Marsh of Khuzestan Province	Mohammadi et al. (2021)
45	*Contracaecum* sp.	*Liza klunzingri*	51	21	Digestive system	N	Coastal waters of BandarAbbass	Nazari et al. (2019)
46	*C. multipapillatum*	*Mesopotamichthys sharpeyi*, *B. grypus*	100, 100	9, 6	Intestine	N	Shadegan Wetland/Ahvaz	Mohammadi et al. (2018)
47	*Contracaecum* sp.	*Nemipterus japonicus*	649	ND	Intestine	N	Persian Gulf/Bushehr	Nematollahi et al. (2018)
48	*Contracaecum* sp.	*Capoeta barroisi*	100	5	Intestine, abdominal cavity	N	Daleki River/Bushehr	Mohajeri Borazjani et al. (2017)
49	*Contracaecum* sp.	*Scomberomorus commerson*, *Otolithes rubber*, *Acipenser persicus*	110, 60, 20	13, 33.3, 10	Abdominal cavity	N	Caspian Sea and Persian Gulf	Adel et al. (2015)
50	*Contracaecum* sp.	*Acanthopagrus latus*	276	12.7	Intestine, abdominal cavity	N	Bahmanshir, Arvand Rud, Hengam and Ahvaz Khuzestan Province	Rasouli (2015)
51	*Contracaecum* sp.	*Otolithes rubber*	60	33.3	Abdominal cavity, digestive system	N	Persian Gulf/Ahvaz, Khuzestan Province	Adel et al. (2014)
52	*Contracaecum* sp.	*C. damascina*	12	8.3	Intestine	N	Kor River basin/Fars Province	Gholami et al. (2014)
53	*Contracaecum* sp.	*B. luteus*, *C. barroisi persica*, *Cyprinion macrostomus tenairidias*, *Chalcolburnus sellal*, *Garra ruffa obtuse*	37, 39, 44, 30, 22	24.32, 12.82, 18.18, 10, 22.72	Digestive system	N	Parishan lake/Kazerun, Fars Province	Golchin Manshadi et al. (2014)
54	*Contracaecum* sp.	*Scomberomorus Commerson*	100	3	Abdominal cavity, muscle	N	Persian Gulf/Bandar Abbas	Adel et al. (2013a)
55	*Contracaecum* sp.	*Pseudorhombus elevates*, *Psettodes erumei*	43, 97	9.27, 16.27	Surface of stomach, abdominal cavity	N	Persian Gulf/Market of Chahbahar	Hosseini et al. (2013)
56	*Contracaecum* sp.	*Clupeonella grimmi*	252	1.6	Abdominal cavity, digestive system, gonads	N	Caspian Sea/Babolsar, Mazandaran Province	Jeddy et al. (2012)
57	*Contracaecum* sp.	*B. sharpeyi*	20	5	Intestine	N	Shadegan Wetland/Susangerd, Khuzestan Province	Rahdar et al. (2012)
58	*Contracaecum* sp.	*B. barbulus*	69	24.63	Digestive system	N	Karoon River/Ahvaz	Zahiri and Razijalali (2012)
59	*Contracaecum* Larvae	Barboid fishes	4	N	Intestine, body cavity	PCR	Parishan Lake, Fars Province	Shamsi and Aghazadeh‐Meshgi (2011)
60	*Contracaecum* sp.	*Chondrostom regium*, *B. grypus*, *B. luteus*	106, 53, 97	11.32, 21.03	Intestine	N	Zohreh/Khuzestan Province	Dadar et al. (2011)
61	*C. osculatum*	*Blicca bjoerkna*	78	17.95	Digestive system	N	Anzali Lagoon, Caspian Sea/Guilan Province	Pazooki et al. (2011a)
62	*Contracaecum* sp.	*Iranocichla hormuzensis*	702	ND	Abdominal cavity	N	Mehran River/Hormuzen, Fars Province	Ansary et al. (2010)
63	*Contracaecum* sp.	*B. grypus*	40	47.5	Abdominal cavity	N	Karkheh River/Khuzestan Province	Mesbah et al. (2010)
64	*Contracaecum* sp.	*Clupeonella cultriventris*, *Clupeonella grimmi*, *Clupeonella engrauliformis*	92, 136, 170	11.8, 5.1, 4.3	ND	N	Caspian Sea/Babolsar, Mazandaran Province	Ghayoumi et al. (2009)
65	*Contracaecum* sp.	*Acanthopagrus bifasciatus*, *Acanthopagrus latus*	40, 40	10, 17.5	Intestine, abdominal cavity	N	Persian Gulf/Ahvaz, Khuzestan Province	Peyghan et al. (2008)
66	*Contracaecum* sp.	*Barbus sharpeyi*, *B.luteus*, *Aspius vorax*, *Liza abu*	96, 175, 44, 130	ND	Intestine, abdominal cavity	N	Hoor‐Al‐Azim, Hoor‐Shadegan and Karoon River, Khuzestan Province	Seyed Mortezei et al. (2008b)
67	*Contracaecum* sp.	*Lutjanus malabaricus*	80, 80, 80	6.25	Abdominal cavity	N	Persian Gulf/Ahvaz, Khuzestan Province	Peyghan et al. (2006)
68	*Contracaecum* sp.	*Barbus grypus*, *Aspius vorax*, *Cyprinus carpio*	701	0.85	Muscle, abdominal cavity	N	Atash, Sobhanieh, Al‐hai and Houfel/Khuzestan Province	Farahnak and Tabibi (2002)
69	*Contracaecum* sp.	*Barbus* sp.	ND	ND	Digestive system	N	Aras River/West Azerbaijan Province	Pazooki and Sayar (2002)
70	*Contracaecum* sp.	*Clupeonella grimmi*	490	N	Abdominal cavity,	N	Caspian Sea	Shamsi et al. (1998a)
71	*Contracaecum* sp.	*Clupeonella engrauliformis, C. grimmi, C. cultriventris*	545, 490, 605	1.3, 1.4, 8.6	Gastrointestinal tract, ovaries, muscles, testis	N	Babolsar Mazandaran Province, Bandar Anzali, Gilan Province	Shamsi et al. (1998b)
72	*Contracaecum* sp.	*Lehias persicus*	2	50	Surface of stomach, intestine, liver	N	Maharlu Lake	González‐Solís et al. (1997)
		*Chalcalhurnus mossulensis*	8	12.5	Surface of stomach, intestine, liver	N	Pulvar River and stream at Naqsh‐e Rostam	
		*C. mossulensis*	7	28.57	Surface of stomach, intestine, liver	N	Pol‐e Berengie at Somduldul	
		*C. mossulensis*	7	14.28	Surface of stomach, intestine, liver	N	Pol‐e Fasa	
73	*C. squalii*	*Acipenseridae fishes*	ND	ND	ND	N	ND	Mokhayer (1974)
74	*C osculatum*	*Esox lucius*	ND	36.7	Digestive system	N	Caspian Sea	Eslami et al. (1972)
75	*Raphidascaroides nipponensis*	*Cynoglossus bilineatus*	100	2	Digestive system, Intestine	N	Persian Gulf	Azodi et al. (2019)
76	*R. acus*	*Esox lucius linnaeus*	30	26.6	Intestine	N	Chamkhale River Mazandaran Province	Sadrinejad et al. (2016)
	*R. acus*	*Esox lucius linnaeus*	30	44.66	Intestine	N	Anzali Wetlands, Mazandaran Province	
	*R. acus*	*Esox lucius linnaeus*	30	10	Intestine	N	Amirkelayeh Wetlands, Mazandaran Province	
77	*Raphidascaris* sp.	*Silurus glanis*	89	29.7	Intestine	N	Amir Kalayeh Wetland/Mazandaran Province	Khara and Sattari (2016)
78	*R. acus*	*Cyprinus carpio*	54	37.04	Intestine	N	Anzali Wetland/Mazandaran Province	Daghigh Roohi et al. (2015)
79	*Raphidascaris* sp.	*Otolithes ruber*	25	12	Abdominal cavity, liver	N	Persian Gulf/Bandarabbas, Hormozgan	Ebrahim Zadeh Mosavi et al. (2015)
80	*R. acus*	*Esox lucius*	120	6.66	Intestine	N	Anzali Wetland/Guilan Province	Fallah et al. (2015)
81	*Raphidascaris* sp.	*Acanthopagrus latus*	276	12.7	Intestine	N	Bahmanshir, Arvand Rud, Hengam and Ahvaz Markets /Khuzestan Province	Rasouli (2015)
82	*R. acus*	*Silurus glanis*	1758	86.05	Intestine	N	Anzali Wetland/Mazandaran Province	Daghigh Roohi et al. (2014)
83	*Raphidascaris* sp.	*Psettodes erumei*	97	15.46	Abdominal cavity	N	Persian Gulf/Market of Chabahar	Hosseini et al. (2013)
84	*Raphidascaris* sp.	*Thunnus tonggol*	100	2	Abdominal cavity, Intestine	N	Persian Gulf/Bandarabbas, Hormozgan Province	Eslami et al. (2011)
85	*R. acus*	*Rutilus frisii kutum*	200	0.5	Abdominal cavity	N	Caspian Sea	Mohammad et al. (2011)
86	*R. acus*	*Blicca bjoerkna*, *Hemiculter leucisculus*	78, 114	1.04, 0.88	Abdominal cavity, intestine	N	Anzali Lagoon, Caspian Sea/Guilan Province	Pazooki et al. (2011a)
87	*R. acus*	*Neogobius melanostomus*, *N. fluviatilis pallasi*, *N. kessleri gorlap*, *N. bathybius*	35, 103, 70, 30	ND	Abdominal cavity	N	Southern part of the Caspian Sea, North of Golestan Province	Pazooki et al. (2011b)
88	*R. acus*	*Esox lucius*, *Scardinius erythrophthalmus*	39, 119	15.4, 20.2	Intestine	N	Boujagh Wetland/Mazandaran Province	Sattari et al. (2011)
89	*Raphidascaris* sp.	*Clupeonella cultriventris*, *Clupeonella engrauliformis*	170, 92	1.2, 1.1	ND	N	Caspian Sea/Babolsar, Mazandaran Province	Ghayoumi et al. (2009)
90	*R. acus*	*Esox lucius*	39	15.38	Digestive system	N	Bojagh Wetland/Giulan Province	Tatina et al. (2009)
91	*Raphidascaris* sp.	*Acanthopagrus latus*	40	30	Intestine	N	Persian Gulf/Ahvaz, Khuzestan Province	Peyghan et al. (2006)
92	*R. acus*	*Rutilus frisii kutum*	30	6.7	Intestine	N	Southern shore of the Caspian Sea	Sattari et al. (2008)
93	*Raphidascaris* sp.	*Pomadasys kaakan*	80	5	Abdominal cavity	N	Persian Gulf, Ahvaz, Khuzestan Province	Peyghan et al. (2006)
94	*R. acus*	*Esox lucius*, *Carassius carassius*, *Abramis brama* *Perca fluviatilis*, *Silurus glanis*, *Tinca tinca*	50, 42, 50, 50, 5	84, 2.4, 10 2.86, 20, 33.33	Intestine	N	Anzali Wetland/Guilan Province	Sattari et al. (2005)
95	*R. acus*	*Esox lucius*, *Tinca tinca*	78, 64	26.9, 5.7	Intestine	N	Amirkelaieh Wetland/Guilan Province	
96	*R. acus*	*Esox lucius*, *Carassius carassius*, *Perca fiuviatilis*, *Silurus glanis*	43, 82, 35, 5	72, 24	Intestine	N	Anzali Wetland in southwest of Caspian Sea	Sattari et al. (2011)
97	*Eustrongylides excicus*	*Neogobius caspius*	170	35.29	Abdominal cavity	N	Chamkhaleh, Kiashahr, Anzali/Giulan Province	Mirnategh et al. (2017)
98	*E. excicus*	*Esox lucius*	30	3.33	Intestine	N	Chamkhale River/Giulan Province	Sadrinejad et al. (2016)
99	*Eustrongylides* sp.	*Alosa saposchnikowii*	30	16.16	Abdominal cavity	N	Southeastern part of the Caspian Sea/Behshahr, Mazandaran Province	Mazandarani et al., 2016)
100	*E. excicus*	*Esox lucius*	120	26.66	Intestine	N	Anzali Wetland/Guilan	Fallah et al., 2015)
101	*E. excicus*	*Silurus glanis*	ND	69.77	Intestine	N	Anzali Wetland/Guilan	Daghigh Roohi et al., 2014)
102	*Eustrongylides* sp.	*Sander lucioperca*	30	23.33	Abdominal cavity	N	Southeastern part of the Caspian Sea/Behshahr, Mazandaran Province	Mazandarani et al. (2014)
103	*E. excicus*	*Acipenser persicus*, *Acipenser stellate*,	8, 17	30.76, 17.64	Digestive system	N	Coasts of Caspian Sea/Mazandaran Province	Khara et al. (2011)
104	*E. excicus*	*Rutilus frisii kutum*	200	1.5	Ventricicle area attached to muscles	N	Southwest part of Caspian Sea	Mohammad et al. (2011)
105	*E. excicus*	*Barbus brachycephalus, B. capito*, *Aspius aspius* *Neogobius fluviatilis*, *Neogobius kessleri*, *Neogobius caspius*	10, 30, 30, 43, 34, 33	50, 40, 33.3, 16.3 50, 18.2	Intestine	N	Southern shore of the Caspian Sea	Sattari et al. (2008)
106	*E. excicus*	*Acipenser gueldenstaedti*, *Acipenser nudiventris*, *Huso huso*	78, 18, 6	15.28, 31.25, 50	Intestine	N	Sefid‐Rud River/Southwest of the Caspian Sea	Sattari and Mokhayer (2006)
107	*E. excicus*	*Esox lucius*, *Perca fluviatilis* *Silurus glanis*	50, 24, 5	6, 24, 20	Intestine	N	Anzali Wetland/Guilan Province	Sattari et al. (2005)
108	*E. excicus*	*Esox lucius*	78	2.6	Intestine	N	Amirkelaieh Wetland/Guilan Province	
109	*E. excicus*	*Neogobius fluviatilis*, *N. kessleri*, *N. caspius*	43, 10, 33	16.28, 50, 18.18	Intestinal	N	Southwest coast of the Caspian Sea	Daghigh Roohi and Satari (2004)
110	*E. excicus*	*Esox lucius*, *Perca fluviatilis*, *Vimba vimba*, *Neogobius kessleri*, *Neogobius fluviatilis*, *Neogobius caspius*	60, 36, 50, 12, 14, 7	5, 33.3, 2.08, 50, 35.7, 14.3	Intestinal	N	Caspian Sea/Giulan Province	Sattari (2004)
111	*E. excicus*	*Clupeonella grimmi*	490	N	Abdominal cavity	N	Caspian Sea	Shamsi et al. (1998a)
112	*Hysterothylacium* sp.	*Sphyraena jello*	150	12	Abdominal cavity, Digestive tract	N	Persian Gulf/Khuzestan, Bushehr and Hormozgan Provinces	Taheri Mirghaed et al. (2016)
113	*Hysterothylacium* larval type VI	*Otolithes ruber*	212	0.47	Intestine	N	Persian Gulf/Bandarabbas, Hormozgan Province	Shohreh and Ghadam (2016)
114	*Hysterothylacium* larval type XV	*Otolithes ruber*, *Psettodes erumei*, *Saurida tumbil*, *Scomberomorus commerson*	120, 120, 120, 120	3.34, 8.34, 25, 0.84	Intestine	PCR	Persian Gulf/Bandarabbas, Hormozgan Province	Shamsi et al. (2016)
115	*Hysterothylacium persicum*	*Scomberomorus commerson*	120	3.34	Intestine	PCR	Persian Gulf/Bandarabbas, Hormozgan Province	
116	*Hysterothylacium* sp.	*Otolithes ruber*	120	1.67	Intestine	PCR	Persian Gulf/Bandarabbas, Hormozgan Province	
117	*Hysterothylacium* larval type VI	*Otolithes ruber*	120	0.84	Intestine	PCR	Persian Gulf/Bandarabbas, Hormozgan Province	
118	*Hy. amoyense*	*Platycephalous indicus*	1	Case report	Gut submucosa	PCR	Persian Gulf/Boushehr Province	Najjari et al. (2016)
119	*Hysterothylacium* type A, B and C	*Saurida tumbil*, *Nemipterus japonicus*	31, 37	ND	Body cavity, ovary	N	Persian Gulf/Choebded, Boushehr Province	Dadar et al. (2016)
120	*Hysterothylacium* type A, B	*Tylosurus crocodilus crocodile*, *Carangoides armatus*	34, 36	ND	Body cavity, ovary	N	Persian Gulf/Choebded, Boushehr Province	
121	*Hysterothylacium* sp.	*Acanthopagrus latus*	276	5.8	Intestine	N	Bahmanshir, Arvand Rud, Hengam and Ahvaz Markets/Khuzestan Province	Rasouli (2015)
122	*Hy. aduncum*	*Neogobius fluviatilis pallasi*, *N. kessleri gorlap*, *N. melanostomus*	103, 70, 35	ND	Intestine	N	Caspian Sea, north of Golestan Province	Pazooki et al. (2011b)
123	*Hysterothylacium* sp.	*Scomberomorus guttatus*	9	11.11	Intestinal	N	Persian Gulf, Shiraz Province	González‐Solís et al. (1997)
124	*Capillaria* sp.	*Pterophyllum scalare*	10	100	Bowel lumen	N	Angelfish stock/Ahvaz, Khuzestan Province	Peyghan et al. (2016)
125	*Capillaria* sp.	*C. damascina*	12	8.3	Abdominal cavity, intestine content	N	River Kor/Khuzestan Province	Gholami et al. (2014)
126	*Capillaria* sp.	*Pterophyllum scalare*	100	18	Digestive tracts	N	Farms of Sari/Golestan Province	Adel et al. (2013b)
127	*Capillaria* sp.	*B. barbulus*	69	2.88	Intestine	N, D	Karoon River/Ahvaz, Khuzestan Province	Zahiri and Razijalali (2012)
128	*Capillaria* sp.	*B. grypus*	40	5	Digestive system, abdominal cavity	N	Karkheh River/Khuzestan Province	Mesbah et al. (2010)
129	*Capillaria* sp.	*Symphysodon aequifasciatus*	ND	ND	Intestine	N	Propagation centres/Tehran	(Rahmati‐Holasoo et al. (2010)
130	*Capillaria* sp.	*C. capoeta gracilis*	14	25	Intestine	N	Hassan Abdali Dam, Zanjan Province	Pazooki et al. (2005)

ND: Not determined, N: Necroscopy, D: Digestion, PCR: Polymerase chain reaction.

**TABLE 5:** Fish‐borne zoonotic acanthocephal in Iran

**Row**	**Parasites**	**Fish species**	**No. exam**	**Prevalence (%)**	**Infected organs**	**Method**	**Lakes, river/area study**	**References**
1	*Corynosoma strumosum*	*Clupeonella grimmi*	60	90	Digestive	N, H	Caspian Sea/Babolsar, Mazandaran Province	Habibi and Shamsi (2018)
2	*C. caspicum*	*Gasterosteus aculeatus*	360	30.3	Intestine, body cavity	N	Caspian Sea/Babolsar, Mazandaran Province	Rahimi‐Esboei et al. (2017)
3	*C. stromosum*	*Esox lucius*	120	6.66	Intestine	N	Anzali Wetland/Guilan Province	Fallah et al. (2015)
4	*C. stromosum*	*Neogobius kessleri gorlap*, *N. melanostomus*, *N. fluviatilis pallasi*	70, 35, 103	3, 2.8, 3.1	Intestine	N	Southern part of the Caspian Sea, North of Golestan Province	Pazooki et al. (2011b)
5	*C. stromosum*	*Clupeonella cultriventris*, *C. engrauliformis*, *C. grimmi*	170, 92, 136	1.8, 16.3, 94.9	Intestine	N	Southern Caspian Sea	Ghayoumi et al. (2009)
6	*C. strumosum*	*Acipenser gueldenstaedti*, *Huso huso*	78, 6	9.72, 33.33	Abdominal cavity	N	Sefid‐Rud River/Southwest of the Caspian Sea	Sattari and Mokhayer (2006)
7	*C. strumosum*	*Gasterosteus aculeatus*	N	April (97.14)	N	N	Gomishan Wetland, Mazandaran Province	Niksirat et al. (2006)
8	*C. stromosum*	*Neogobius fluviatilis*, *N. kessleri*, *N. melanostomus*	43, 14, 10	20.93, 86, 10	Intestine	N	Southwest coast of the Caspian Sea	Daghigh Roohi and Satari (2004)
9	*C. stromosum*	*Neogobius kessleri*	11	10	Abdominal cavity	N	Southwest coast of the Caspian Sea	Saeedi and Fani saberi (2003)
10	*C. stromosum*	*Clupeonella grimmi*	490	N	Abdominal cavity	N	Caspian Sea	Shamsi et al. (1998a)
11	*C. stromosum*	*Clupeonella engrauliformis*, *C. grimmi, C. cultriventris*	545, 490, 605	15.3, 91.3, 4.3	Gastrointestinal tract, ovary, testis	N	Babolsar Mazandaran Province, Bandar Anzali, Gilan Province	Shamsi et al. (1998b)

ND: not determined, H: histopathology.

**Impacts**

- The potential risk factors for fish‐borne parasitic zoonoses are consumption of raw infected fish and fish products and contact with contaminated water or infected fish.
- The presence of zoonotic parasites in fish in Iran could impose a potential risk to human health.
- It was documented that among the pathogenic parasites infecting fish, majority of them are zoonotic and the most common fish‐borne zoonotic parasites were the protozoa, trematodes, cestodes and nematodes

## 3 FISH‐BORNE ZOONOTIC PARASITES

A total of 200 documents were obtained with references to FBZPs in Iran. These result show that many studies have been conducted on the parasitic infections of fish in Iran; however, from the point of view of fish parasites with zoonotic potential, there is no comprehensive review with many studies focused on individual fish species or parasites. During this study, according to the available data, 58 species of zoonotic parasites were recorded from 112 fish species, from different faunal regions. Protozoa had the most parasites (36 species) followed by nematodes, trematodes, cestodes and acanthocephalan 16, 2, 2 and 2 different species of parasites, respectively.

### 3.1 Fish‐borne zoonotic protozoa

Three protozoa in fish were identified in the literature, *Balantidium* and *Sarcocystis*, with the first in *Barbus sharpeyi*, *Barbus grypus* and *Ctenopharyngodon idella* and the latter only in *B. sharpeyi*.

#### 3.1.1 *Balantidium* spp

The genus *Balantidium* has a large number of species that have been reported in the digestive tracts of a widely diverse range of invertebrate and vertebrate hosts such as molluscs, arthropods, fish, amphibians, reptiles, birds and mammals including humans (Biswas & Mukherjee, 1981; Bradbury, 1994a, 1994b; Li et al., 2008, 2014). The protozoa reside in the intestine and are considered either commensal or endosymbionts in fish with no indications that those in fish infect humans; however, they are included in this review due to the need for a better overall understanding of the species present. *Balantidium coli* is the species infecting humans, non‐human primates and swine and can be either asymptomatic or cause diarrhoea. In fish, 13 species have been reported in freshwater fish, eight of which have been described in China and two of which (*Balantidium ctenopharyngodoni* and *B. polyvacuolum*) are reported most frequently (Chen, 1955; Li, 1963; Li et al., 2014; Zhao & Ma, 1992). Four species have been reported in marine fish with *Balantidium prionurium* reported more commonly particularly in surgeonfish (*Prionurus punctatus*) (Norman Grim, 1985).

Data collected from different regions of Iran showed that this parasite was found in two species of fish (*Barbus sharpeyi* and *Barbus grypus*) from Shadegan and Sosangerd city and Khouzestan Province (Rahdar et al., 2012; Seyed Mortezaei et al., 2008) (Table 1).

#### 3.1.2 Sarcosystis

For the more than 150 species *of Sarcocystis*, most intermediate hosts include herbivorous mammals and humans and other primates but also some birds, reptiles and possibly fish. Definitive hosts include carnivores or omnivores, including humans and some reptiles and raptorial birds (Fayer et al., 2015). Final hosts transmit the infection to intermediate hosts via the faecal–oral route and final hosts become infected via the consumption of intermediate hosts. Intermediate hosts also can become infected by consuming the cystic stage (sarcocyst) in muscle in another intermediate host. Humans can be definitive hosts for some species and dead‐end intermediate hosts for other species, becoming infected with *Sarcocystis* via ingestion of water or food with sporocysts (excreted by carnivorous definitive hosts) or eating raw/undercooked meat containing a sarcocyst (Rosenthal et al., 2012). Symptoms in humans can include nausea, vomiting and enteritis (acute, chronic and severe) (Fayer et al., 2015).

The clinical significance of *Sarcocystis* in fish as well as the role of fish in contributing to zoonosis are not well understood. Most reports in fish have been in trout. The first report of *Sarcocystis* infection (15%) in muscles of *Barbus sharpeyi* from Shadegan and Susangerd city, Iran was by Rahdar et al. (2012). They suggested that consumption of raw or under cooked fish in the endemic region be avoided (Table 1).

#### 3.1.3 *Myxobolus* spp

Myxozoans are economically important group of microscopic metazoan parasites, which cause disease in a large variety of commercially important fishes. They have also been reported in platyhelminthes, reptiles, amphibians, mammals as well as in faecal sample of human beings (Boreham et al., 1998). Among myxosporeans, the genus *Myxobolus* includes the highest number of species. In Iran till now, altogether 43 different *Myxobolus* spp. have been reported (Masoumian et al., 2007). The highest frequency of *Mycobulus* spp. was related to *M. dispar* with a prevalence of 100% from *Aspius aspius taeniatus* in the Aras and Mahabad Dams, Western Azerbaijan Province, followed by *M. musayevi* with prevalence of 60% and *M. cristatus* with a prevalence of 59.5% from *Capoeta damascina* in Halil‐Rud River, Kerman Province (Table 1).

### 3.2 Fish‐borne zoonotic trematodes

#### 3.2.1 Heterophyes heterophyes

*Heterophyes heterophyes* is a small parasitic fluke that infects humans who eat raw or undercooked fish infected with the metacercaria stage of the parasite. A variety of mammals (e.g., dogs, foxes and jackals) other than humans take the role of reservoir hosts (Chai, 2007). The first intermediate host of *H. heterophyes* are snails, *Cerithidia* spp. and *Pironella* spp., which are found in Asia and the Middle East, respectively. *H. heterophyes* is commonly found in the Middle East, Philippines, Taiwan, Korea, China and Japan (Chai, 2007; Yu et al., 1994). Massoud et al. (1981) showed that the rate of infection with *H. heterophyes* in man and carnivores in Khuzestan, Iran was 8% and 9.5% respectively (Massoud et al., 1981). Farahnak (2001) identified a prevalence of 1.9% in people of Mazreeh district in Khouzestan Province, Iran (Farahnak, 2001). Carnivores, particularly golden jackals, foxes and dogs serve as the main natural reservoir host for heterophyid infection in the Khuzcstan area, Iran. These animals frequently feed on frogs, brackish‐water fish, snails and other small creatures in the area and contaminate the water bodies with their faeces, so that aquatic snails become infected and transmission continues. Prevalence in fish in Iran has not been determined.

#### 3.2.2 Clinostomum complanatum

*Clinostomum complanatum* is a digenetic trematode, which usually infects birds after consuming infected fish or amphibians. Metacercariae of *C. complanatum* are often yellow and encyst in a variety of sites in the body such as the oral cavity, gills, intestines, tail, muscles and eye sockets of fish or amphibians, causing ‘yellow grub disease’. According to previous epidemiological studies, 12 species of freshwater fish were recorded as the second intermediate hosts (Chung et al., 1995; Rim et al., 1996), and one species of freshwater snail (Species) was reported as the first intermediate host of *C. complanatum* (Chung et al., 1998). If a human consumes an infected raw fish, the fluke can attach to the surface of the mucus membrane of the throat and cause a clinical syndrome called halzoun. Several reports are available on the occurrence of *C. complanatum* in Iranian freshwater fish in different areas of Iran. The results showed that the highest infection with *C. complanatum* (61.4% and 28%) is observed in gill cavities, gill arches, operculum, muscles and fins of *Capoeta capoeta gracilis* from Shirood River, Mazandaran (Ghazifard et al., 2011; Roohi & Malek, 2005). Infection with *C. complanatum* has been observed in several other fish species in Iran with prevalence ranging from 3% to 16.6% (Table 2). This difference may be due to the climatic conditions of the study area, species, length and habitat (lentic or semi‐lotic) of the host fish (Dias et al., 2006). Dias et al. (2006) showed that there are host fish preferences and a strong and positive effect of the host length upon the prevalence, that is, the larger the fish, the higher the probability of being infected (Dias et al., 2006). Given the prevalence of *C. complanatum* and variety of fish infected, prevalence studies in humans are needed and awareness of halzoun increased.

### 3.3 Fish‐borne zoonotic cestodes

#### 3.3.1 Ligula intestinalis

*Ligula intestinalis* is a cestode from the family pseudophilidae, which are three‐host parasites. It infects a range of fish species and is found in free living and farmed fish all over the world (Scholz et al., 2003) and there are several reports of human infestations with this parasite (Urdes & Hangan, 2013). Ligula has a life cycle containing copepods as first, fish as second and piscivorous birds as the final hosts. Parasite eggs are released via bird faeces into the water and develop to ciliated coracidium larva. It survives 1–2 days in the water and develops to procercoid larva in copepod. Fish eat the copepod and subsequently, a large plerocercoid larva develops in the body cavity of the fish (İnnal et al., 2007). In Iran, the highest overall prevalence of ligulosis was recorded in *Chalcalburnus chalcoides* (83.08%) (Garedaghi & Mohammadi, 2012), *Alburnus filippi* (81.87%) and *Alburnoides biponctatus* (80%) (Mortazavi et al., 2005) (Table 3). The difference in the prevalence of this parasite in different regions of Iran is attributed to the ecosystem of the region. In basins such as Vahdat, Aras, Sattar Khan and Zayandehrud dams compared to Gorganrood and Algol, which have more water, there is a larger population of fish‐eating birds, which play an important role in the spread of this parasite (Mazandarani et al., 2018). In contrast to trematodes such as *C. complanatum*, the prevalence of *L. intestinalis* decreases with increasing fish size, probably due to selective mortality among parasitised individuals (Garedaghi & Mohammadi, 2012; Mortazavi et al., 2005).

#### 3.3.2 *Dibothriocephalus latus* (formerly *Diphyllobothrium latum*)

*Dibothriocephalus latus* (formerly *Diphyllobothrium latum*), or broad fish tapeworm, is one of the pseudophyllidean cestodes transmitted via aquatic species. The parasite has a complex life cycle with piscivorous animals, including man, being the final host. There are two intermediate hosts: a copepod as the first intermediate host and a predatory freshwater, anadromous or marine fish as the second intermediate host. Human infection with *D. latum* is acquired by eating uncooked freshwater fish containing the parasite's plerocercoid cysts (Dick, 2007). Human infections can be asymptomatic or cause diarrhoea, abdominal pain and anaemia (Santos & Howgate, 2011). The first report of *D. latum* in fish in Iran was by Mokhayer in 1981 who identified it in *Barbus brachycephalus* (Mokhayer, 1981). Later, Moghainemi (1996) found the plerocercoid stage in *Barbus sharpeyi*, *Barbus grypus* and *Planiliza abu* from Hoor‐Alazim Lagoon, Khuzestan Province, Iran (Moghainemi, 1996).

### 3.4 Fish‐borne zoonotic nematodes

#### 3.4.1 *Pseudoterranova* spp

*Pseudoterranova* is a genus of parasitic nematodes within the family Anisakidae. The lifecycle of *Pseudoterranova* spp. involves marine mammals such as sea lions, seals and walruses as final hosts (Hochberg et al., 2010) and planktonic or benthic crustaceans as intermediate hosts. Fish are a second intermediate or paratenic host (Brunet et al., 2017). In some regions, increasing seal numbers have prefaced an increase in fish infected with *P. decipiens* (Lunneryd et al., 2015). *Pseudoterranova*, which is found in wild caught and farmed fish, can impact the health and swimming ability of infected fish, resulting in mortality (Buchmann & Mehrdana, 2016). Species belonging to this genus also can adversely impact human health with exposure occurring via the consumption of raw or under cooked infected fish (Shamsi & Sheorey, 2018; Shamsi & Suthar, 2016). Cases of human infection have been reported from consuming partially cooked fish infected with *P. decipiens* (Shamsi, 2019), *P. cattani* (Weitzel et al., 2015) and *P. azarasi* (Arizono et al., 2011).

*Pseudoterranova* was detected from *Saurida tumbil* (25%) and *Tylosurus crocodilus crocodile* (14.28%) in the Persian Gulf, Iran by Dadar et al. (2016). In a study by Hosseini et al. (2013), this parasite was found in the stomach of *Psettodes erumei* from the Persian Gulf with a prevalence of 4.1% (Hosseini et al., 2013). The species of this parasite infecting fish from Iran needs to be determined to better understand the health risk to fish and people.

#### 3.4.2 *Anisakis* spp

Anisakiasis is the zoonotic disease triggered by the third stage larvae of the nematodes, *Anisakis* (Nieuwenhuizen & Lopata, 2013). This parasite habitually infects adult marine mammals. Intermediate and/or paratenic hosts include crustaceans, cephalopods and fish (Nieuwenhuizen & Lopata, 2013). Humans are accidentally infected via the ingestion of raw or inadequately cooked or fish and shellfish (Aibinu et al., 2019). Symptoms of acute anisakiasis include severe abdominal pain, nausea and vomiting. Some of these symptoms closely mimic peptic ulcer, appendicitis or peritonitis with the most concerning presentation being allergic sensitisation, which is usually serious and ranges from urticaria to anaphylactic shock (Villazanakretzer et al., 2016).

*Anisakis simplex*, the primary zoonotic species, was believed to occur mainly in spotted chub mackerel (*Scomber japanicus*) and Japanese flying squid (*Todarodes pacificus*) (Nagasawa & Moravec, 1995). However, more recently, Abollo et al. (2001) concluded that most species of cephalopods and fish can potentially harbour these marine parasitic nematodes as 200 fish and 25 cephalopods species have been identified as hosts for *Anisakis*.

In Iran, the highest and lowest rate of infection with *Anisakis* spp. was observed in Long Tail Tuna (*Thunnus tonggol*) (89%) (Eslami et al., 2011) and *Clupeonella grimmi* (0.4%) (Jeddy et al., 2012), respectively with *Acipenser* spp. and *Barbus* spp. also commonly infected (Table 4). In many studies conducted in Iran, the species of *Anisakis* has not been determined; to better understand risk of human infection, more speciation is needed. No human *Anisakis* infections have been reported in Iran.

#### 3.4.3 *Contracaecum* spp

*Contracaecum* spp. are another genus of zoonotic nematodes from the family Anisakidae. In their life cycle, *Contracaecum* spp. infect marine mammals and piscivorous birds as final hosts and crustaceans and a wide range of fish species as their intermediate hosts (Anderson, 2000). *Contracaecum* spp. larvae can infect humans with the disease referred to as anisakiasis, the same name as used to refer to disease caused by *Anisakis* spp. As with *Anisakis* spp., infection in humans with *Contracaecum* spp. occurs via the consumption of raw or undercooked fish which are host to the third stage larvae. The symptoms of anisakiasis include abdominal pain and distention, diarrhoea with blood and mucus, nausea and a mild fever. There can also be allergic reactions such as rash and itching, and occasionally there can also be anaphylaxis (Ivanović et al., 2017).

The first report of *Contracaecum osculatum baicalensis* in Iran was by Eslami et al. (1972) who documented the parasite in the gastrointestinal tract of pike (*Esox lucius*; from the Caspian Sea with a prevalence of 36.7%) (Eslami et al., 1972). In subsequent studies, *Contracaecum squalii* was found in acipenseridae fish (Mokhayer, 1974). *Contracaecum* spp. have now been recorded from several fish species in Iran (Table 4). The infection of *Contracaecum* sp. ranges from 47.4% in *Barbus Grypus* from Karkheh River, Khuzestan (Mesbah et al., 2010) to 0.14% in *Cyprinus carpio* from Lagoons including Atash, Sobhanieh, Al‐hai and Houfel, Khuzestan Province (Farahnak & Tabibi, 2002). The infection has been associated with low temperature in sampling sites and cooler months. Moreover, environmental changes such as water and air temperature, salinity and level of dissolved oxygen can cause the fish body weakness, increasing the risk of parasitism (Gholami et al., 2014).

#### 3.4.4 *Raphidascaris* spp

The genus Raphidascaris belongs to the Anisakidae family and occurs in a variety of fish in Europe, Asia and North America. The parasite uses invertebrates as a paratenic host, fish as the intermediate host and piscivorous fish as the definitive host (Jahantab et al., 2014). There are some reports on its zoonotic importance (Doupé et al., 2003), but according to Cheng (1998) since the adults of *Raphidascaris* are intestinal parasites of fish, it is doubtful whether they can cause human infection (Cheng, 1998). However, the collected data showed that this parasite was isolated from freshwater fish in Iran with a prevalence of 0.5% in *Rutilus frisii kutum* to 86.05% in *Silurus glanis* (Daghigh Roohi & Satari, 2004). *Raphidascaris* spp. including *R. acus* causes liver pathology, poorer condition, mortality in yellow perch (*Perca flavescens*), its intermediate host and other fishes and economical loses (Kotob et al., 2016; Marcogliese et al., 2005; Szalai & Dick, 1991).

#### 3.4.5 *Hysterothylacium* spp

*Hysterothylacium* is a genus of parasitic roundworms in the family raphidascarididae. As of 2020 it contains of over 70 species and is considered one of the largest of the ascaridoid genera parasitising fish (Ghadam et al., 2018; Li et al., 2007; Shamsi, 2017). The life cycle involves predatory teleost fish as the final host and various species of invertebrates and teleost as the intermediate hosts (Deardorff & Overstreet, 1980). In general, these are not considered a hazard for humans; however, some species are potentially zoonotic (Andrade‐Porto et al., 2015; Deardorff & Overstreet, 1981). The intensity of the infection with this parasite has been correlated with the size of the host with larger hosts having heavier infections (Aloo‐Obudho et al., 2004; Taheri Mirghaed et al., 2016). Shamsi et al. (2016) reported *H. persicumin* and *Hysterothylacium* larval type XV in *Saurida tumbil* fish from the Persian Gulf with prevalence of 25% with other studies reporting lower infection rates (0.84%) (Shamsi et al., 2016). As with many other fish parasites, a better understanding of the species present and the relation of these species to zoonosis is needed. *Hysterothylacium* species can affect the health of the host fish which could lead to their death, with a notable economic outcome (Li, 2008)

#### 3.4.6 *Eustrongilides* spp

*Eustrongylides* spp. is considered a freshwater fish nematode with a complex life cycling including birds as a final host, oligochaetes and fish as first and second intermediate hosts, and other fish and reptiles being paratenic hosts. Humans who consume raw or undercooked freshwater fish containing larval stages of this parasite can develop eustrongylidosis which includes gastritis and intestinal perforation (Branciari et al., 2016; Ljubojevic et al., 2015a). Some of the more commonly infected fish species include *Neogobius fluviatilis*, *N. kessleri*, *N. caspius* and *Silurus glanis* (14.3%–69.77%) (Daghigh Roohi et al., 2014; Sattari et al., 2002, 2008), The diversity of fish species infected with *Eustrongylides excisus* (L) can be high, but the intensity and abundance of the parasite low in individual fish (Sattari et al., 2002). The diversity of the final and first second intermediate hosts and the existence of large populations of *N. fluviatilis*, *N. kessleri* and *N. caspius* in the Caspian Sea and other areas studies in Iran likely contribute to the high prevalence seen.

#### 3.4.7 *Capillaria* spp

More than 250 species of *Capillaria*, which belong to the nematode superfamily trichinelloidae, are known to infect vertebrates. Of these, *Capillaria philippinensis*, a fish‐borne zoonotic parasite, is of medical concern (El‐Dib et al., 2015). The life cycle of *Capillaria* involves fish as intermediate hosts and fish‐eating birds (Piscivorous) as final hosts. Ingestion of raw or undercooked fish results in infection of humans, where they reside in the intestine, burrowing in the mucosa. The female adult worms produce unembryonated eggs, some of which can embryonate in the intestine, releasing larvae and causing autoinfection. The first human case of infection related to the consumption of raw or undercooked fish was detected in the Philippines in 1963 (Chitwood et al., 1999). In Iran, the first human reports of *C. philippinensis* were reported by Aftandelians et al. (1977) followed by reports by Hoghooghi‐Rad et al. (1987) and Aghdam et al. (2015). Several species of fish in Iran have been identified with zoonotic species of Capillaria including *Capoeta damascina* (8.3%) (Gholami et al., 2014), *Barbus grypus* (5%) (Mesbah et al., 2010), *Pterophyllum scalare* (Peyghan et al., 2016), *Symphysodon aequifasciatus* (Rahmati‐Holasoo et al., 2010), *Pterophyllum scalare* (18%) (Adel et al., 2013b) and *Barbus barbulus* (2.88%) (Zahiri & Razijalali, 2012).

### 3.5 Fish‐borne zoonotic acanthocephalan

#### 3.5.1 *Corynosoma* spp

Acanthocephalans of the genus *Corynosoma* are zoonotic parasites that amphipod crustaceans, pinnipeds and fish serve as intermediate, final and paratenic hosts, respectively. Humans are infected by eating infected fish (Sasaki et al., 2019). It has been generally assumed that humans are an unsuitable host for *Corynosoma*, because only one case of infection with immature *Corynosoma strumosum* has been reported from Alaska (Schmidt, 1971). However, recent reports of human corynosomiasis from Japan showed that intestinal adults can reach sexual maturity (i.e. laying eggs), suggesting humans to be a suitable final host (Fujita et al., 2016; Takahashi et al., 2016). The species composition of *Corynosoma* in fish varies according to sea areas with *C. strumosum* tending to be the primary species (Sinisalo & Valtonen, 2003).

*Corynosoma strumosum* are common in commercial fish species in Iran (Sasaki et al., 2019). The highest prevalence of infection with *C. strumosum* was observed in *Casterosteus aculeatus* (97.14%) (Niksirat et al., 2006), followed by in *Clupeonella grimmi* (94.9%) (Ghayoumi et al., 2009). However, the existence of uninfected fish species indicates that the Corynosoma infections fluctuate perhaps due to habitats and food habits of fish (Sasaki et al., 2019).

## 4 CONCLUSION

Changes in food habits/tastes in recent years have led to an increase in the consumption of raw fish and less cooked fish products and this new tendency has increased the risk of exposure of the consumer to parasitic hazards. In conclusion, this review revealed that some of the fish‐borne zoonotic parasites are common in different freshwater fish species mainly *Barbus* spp., *Capoeta* spp., *Cyprinus* spp. and *Neogobius* spp. in different regions of Iran. Fish‐borne zoonotic parasites identified include nematodes (*Anisakis* spp., *Pseudoterranova* spp., *Raphidascaroides* spp., *Contracaecum* spp., *Eustrongylides* spp., and *Capillaria* spp.), trematodes (*Clinostomum complanatum* and *Heterophyes heterophyes*), cestodes (*Ligula intestinalis* and *Diphyllobothrium latum*) and protozoa (*Balantidium* spp. and *Sarcosystis* sp.).

Despite the fact that fish‐borne parasitic zoonosis due to the culture and food habits in Iran and the lack of consumption of raw fish products has not been reported, but due to eating raw or undercooked meat of infected fish or contact with contaminated water or infected fish cause human infection, so control methods such as complete cooking of fish meat and avoiding contact with potentially contaminated water are suggested. Awareness‐raising activities should also be conducted on the nature of fish‐borne zoonotic parasites and the risk of consuming raw fish.

## AUTHOR CONTRIBUTIONS

Nasser Hajipour: conceptualisation; data curation; investigation; methodology; supervision; writing – original draft; writing – review & editing. Hadi Valizadeh: investigation. Jennifer Ketzis: conceptualisation; investigation; methodology; writing – original draft; writing – review & editing.

## CONFLICT OF INTEREST

There is no conflict of interest.

### ETHICAL STATEMENT

Systematic reviews generally do not need ethics committee or institutional review board approval.

### PEER REVIEW

The peer review history for this article is available at https://publons.com/publon/10.1002/vms3.981.

## Data Availability

Research data are not shared.
